# How Task Goals Mediate the Interplay between Perception and Action

**DOI:** 10.3389/fpsyg.2013.00247

**Published:** 2013-05-07

**Authors:** Pascal Haazebroek, Saskia van Dantzig, Bernhard Hommel

**Affiliations:** ^1^Cognitive Psychology, Institute of Psychology, Leiden UniversityLeiden, Netherlands; ^2^Department of Brain, Body and Behavior, Philips ResearchEindhoven, Netherlands

**Keywords:** stimulus–response congruency, task set, perception–action interaction, Wii balance board, connectionist modeling, Simon effect, top-down modulation

## Abstract

Theories of embodied cognition suppose that perception, action, and cognition are tightly intertwined and share common representations and processes. Indeed, numerous empirical studies demonstrate interaction between stimulus perception, response planning, and response execution. In this paper, we present an experiment and a connectionist model that show how the Simon effect, a canonical example of perception–action congruency, can be moderated by the (cognitive representation of the) task instruction. To date, no representational account of this influence exists. In the experiment, a two-dimensional Simon task was used, with critical stimuli being colored arrows pointing in one of four directions (backward, forward, left, or right). Participants stood on a Wii balance board, oriented diagonally toward the screen displaying the stimuli. They were either instructed to imagine standing on a snowboard or on a pair of skis and to respond to the stimulus color by leaning toward either the left or right foot. We expected that participants in the snowboard condition would encode these movements as forward or backward, resulting in a Simon effect on this dimension. This was confirmed by the results. The left–right congruency effect was larger in the ski condition, whereas the forward–backward congruency effect appeared only in the snowboard condition. The results can be readily accounted for by HiTEC, a connectionist model that aims at capturing the interaction between perception and action at the level of representations, and the way this interaction is mediated by cognitive control. Together, the empirical work and the connectionist model contribute to a better understanding of the complex interaction between perception, cognition, and action.

## Introduction

Theories of embodied cognition (e.g., Glenberg, 1997; Barsalou, 1999; Wilson, 2002) suggest that cognition, perception, and action are tightly intertwined and share common representations and processes. In the last decade, this view has been studied extensively, and much evidence in its favor has been accumulated. Many studies have demonstrated that cognition interacts with perception and action, suggesting that these systems share the same representations and processes (e.g., Pecher and Zwaan, 2005). In this study we particularly focus on how cognition can modulate the interaction between perception and action by assessing the role of task instruction on automatic processes in stimulus–response translation. This interaction is demonstrated in an empirical study and further explained by simulations using a connectionist model (HiTEC, Haazebroek et al., submitted). We first describe bilateral interactions between perception, cognition, and action and subsequently focus on the influence of task context on the interaction between perception and action.

### Interactions between perception, cognition, and action

The interaction between perception and cognition can be demonstrated by so-called spatial congruency effects. Several studies have found interactions between the meaning of words and the spatial position of those words on the computer screen. For example, people respond faster to a word such as *helicopter* or *stork* when it is presented at the top of the computer screen than when it is presented at the bottom of the screen (Šetic and Domijan, 2007). Other studies showed that the spatial meaning of a word may attract attention to a particular location on the screen (e.g., Estes et al., 2008; Zanolie et al., 2012). Spatial congruency effects are also found with words referring to abstract concepts that are metaphorically connected to spatial locations, such as power (Schubert, 2005; Zanolie et al., 2012), valence (Meier and Robinson, 2004), divinity (Meier et al., 2007), or magnitude (Fischer et al., 2003; Pecher and Boot, 2011), but see Lakens (2012) for an alternative explanation, based on polarity correspondence. Furthermore, studies have shown that perceiving motion in a particular direction interacts with the processing of sentences or words describing motion in the same direction (e.g., Kaschak et al., 2005; Meteyard et al., 2007, 2008).

Likewise, spatial congruency effects also occur in the interaction between cognition and action. For example, participants are faster to respond to a sentence when the direction of the response matches the direction of the action described in the sentence. This so-called *action compatibility effect* (see Zwaan and Yaxley, 2003; Zwaan et al., 2012) has been found with different kinds of movement, such as moving the hand toward or away from the body (Glenberg and Kaschak, 2002) and rotating the hand (Zwaan and Taylor, 2006). These results are taken as evidence that the representations underlying conceptual processing partially overlap with the representations underlying the preparation and execution of action.

Finally, spatial congruency effects occur in the interaction between perception and action. Much research has been devoted to stimulus–response congruency (SRC) effects; the canonical example being the Simon effect (Simon and Rudell, 1967; Hommel, 2011). In the typical Simon task, stimuli vary on a spatial dimension (e.g., randomly appearing on the left or right) and on a non-spatial dimension (e.g., having different colors). Participants have to respond to the non-spatial stimulus feature by performing a spatially defined response (e.g., pressing a left or right key). Although the location of the stimulus is irrelevant for the response choice, it nevertheless influences the response time and accuracy, suggesting interaction between stimulus perception and response planning. Participants respond faster (and more accurately) when the stimulus location is congruent with the response location than when the stimulus location is incongruent with the response location. The Simon effect has been replicated numerous times and has been used frequently as a methodological tool to investigate perception, action, and cognitive control (for an overview, see Hommel, 2011).

### Influence of cognitive control on the interplay between perception and action

To account for SRC effects, traditional cognitive theories, and computational models of stimulus–response translation typically assume that: (1) responses are represented by spatial codes (e.g., Wallace, 1971), (2) attending to a stimulus automatically produces a spatial stimulus code, and (3) the outcome of a comparison between the spatial stimulus code and the spatial response code produces the compatibility effect. Crucially this comparison is assumed to occur automatically and arise from the fact that stimuli and responses are similar (e.g., have dimensional overlap, Kornblum et al., 1990, 1999; but see Proctor and Lu, 1999; Tagliabue et al., 2000 for accounts based on over-learning). Indeed, in typical computational models of SRC effects, such as the Simon effect, stimuli are represented in terms of non-spatial task-relevant codes (e.g., “red shape” and “blue shape”) and spatial task-irrelevant codes (e.g., “left shape” and “right shape”), and responses are also represented in terms of spatial codes (e.g., “left key” and “right key”). Stimulus codes and response codes are connected using two routes (e.g., Kornblum et al., 1990; De Jong et al., 1994; Zorzi and Umiltà, 1995). A direct route connects the spatial stimulus codes to the corresponding spatial response codes, which is assumed to reflect the automatic process. The task instruction (e.g., “*when you see a red shape, press the left key*”) is implemented as a soft-wired connection from the non-spatial stimulus code (e.g., “red shape”) to a spatial response code (e.g., “left key”), following the task instruction. This is assumed to reflect the controlled process. Now, when a compatible stimulus is presented (e.g., a red shape presented on the left), both the hard-wired spatial connections and the soft-wired task instruction-based connections contribute to a speedy activation of the correct response code. Conversely, when an incompatible stimulus is presented (e.g., a red shape presented on the right), the direct route activates the incorrect response. The controlled route, however, activates the response determined by the task instruction, which eventually wins the competition. As a result, processing incompatible stimuli results in longer reaction times than processing compatible stimuli. In sum, the stimulus–response congruency effect arises from the interplay between the direct route, reflecting automatic comparison between spatial stimulus and response codes, and the controlled route, reflecting the task instructions.

However, the various spatial congruency effects mentioned in Section “Interactions Between Perception, Cognition, and Action” also suggest an interaction between cognition and perception and between cognition and action. Hence, it is to be expected that the (cognitive) task set may influence the automatic translation from spatial stimulus codes to spatial response codes. Indeed, various studies have demonstrated that SRC effects are strongly influenced by the task. For instance, Riggio et al. (1986) reported that when participants responded with sticks that were either parallel or crossed, the Simon effect was found to relate to the stick end position, not to the hands holding the sticks. In a study by Guiard (1983), participants had to respond with a steering wheel. Their results suggest that not the position of the hands but the steering direction (as in a car) determines the Simon effect, indicating an even more abstract notion of left or right responses. It is this task- and intention-dependent left-ness or right-ness, rather than the actual physical location of a response, that seems to interact with the spatial location of the stimulus and thereby yields the Simon effect – an argument that can also be made for other stimulus–response effects (Hommel, 2000).

In a study by Hommel (1993), the role of task instruction was assessed empirically. Hommel had participants responding with left and right keypresses to the high vs. low pitch of tones, respectively. As usual in a Simon task, the tones randomly appeared on the left or right side. Importantly, when a key was pressed a light flashed on the opposite side of the keypress, which allowed instructing participants in two different ways: one group of participants was instructed to “*press the left/right key*” in response to the pitch of the tone, whereas another group was instructed to “*flash the right/left light*.” Given the wiring of lights to response keys, all participants carried out exactly the same movements in response to the same stimuli, but they did so for different reasons: one group in order to press the keys and the other in order to flash the lights. Whereas the Key group showed a standard Simon effect with faster responses when the tone location and key location corresponded, the Light group showed the opposite effect: faster responses when the tone location and light location corresponded. The fact that the irrelevant stimulus locations had an effect at all suggests that stimulus locations were processed and cognitively coded, and that they interacted with spatial response codes. However, the observation that the impact of this interaction on behavior was determined by the instruction and, thus, by the goal representation this instruction must have established, suggests that the interplay between perception and action is controlled by task goals.

Addressing the role of task goals in SRC, Ansorge and Wühr (2001) formulated the response-discrimination hypothesis that states that response representations are not automatically formed, but rather top-down controlled. Only spatial features that discriminate between alternative responses are represented and thus give rise to a Simon effect. This resonates with the conclusions in a general review by Proctor and Vu (2006) that the Simon effect is not resulting from an automatic activation of a corresponding response by means of a hard-wired (e.g., Kornblum et al., 1990) or over-learned (e.g., Umilta and Zorzi, 1997) route; rather the task defines S–R associations that mediate this responding.

### HiTEC

Although it is clear that task context influences SRC, and several hypotheses have been suggested, an overarching framework that connects the different findings and explains computationally how perception, action, and cognition interact *in terms of neurally plausible representations and processes* is still lacking. The development of computational models is mentioned as one of the main challenges for the field of embodied and grounded cognition (Barsalou, 2008, 2010; Borghi and Pecher, 2011; Pezzulo et al., 2011).

To address this challenge, we developed HiTEC, a connectionist computational cognitive model that aims at capturing the interaction between perception and action in terms of neurally plausible representations and processes, and the way this interaction is mediated by cognitive control (Haazebroek et al., 2011, submitted). HiTEC is meant to be a connectionist model that is plausible in terms of neural processing properties and global cortical connectivity. HiTEC enables simulation of human perception and action control, based on the principles and assumptions of the Theory of Event Coding (TEC; Hommel et al., 2001).

Theory of event coding is a general theoretical framework that addresses how perceived events (i.e., stimuli) and produced events (i.e., actions) are cognitively represented and how their representations interact to generate perceptions and action plans. According to TEC, stimuli, and actions are represented in a common representational format, using *the same* feature codes. These codes refer to the distal features of objects and events in the environment, such as shape, size, distance, and location, rather than the proximal features that are registered by the senses. For example, a stimulus presented on the left and an action performed on the left both activate the same distal code representing “left.” It is theorized (Hommel et al., 2001) that feature codes emerge from regularities in sensorimotor experience and that they can also be activated conceptually (e.g., by means of verbal labels, Hommel and Elsner, 2009). When a stimulus (or action–effect) is registered, it is represented by *sensory* *codes* that in turn activate associated distal *feature codes*.

Theory of event coding stresses that perception and action are flexible; that is, they are tuned to the current context and are subject to cognitive control (Hommel et al., 2001). Codes are “intentionally weighted”; the strength of their activation depends on the task context (Memelink and Hommel, 2012). Feature dimensions that are relevant for the task at hand are weighted more strongly than irrelevant dimensions. For example, if the task is to grasp an object, feature dimensions that are relevant for grasping (such as shape, size, location, and orientation) will be enhanced, so that object features on these dimensions have more influence on processing than feature dimensions that are irrelevant for grasping (e.g., color or sound; Fagioli et al., 2007).

Importantly for the present study, intentional weighting can also affect the coding of response representations. In Hommel (1993) it can be argued that the task set results in stronger weighting of key vs. light location, depending on the instruction. One could ask, however, whether this implies weighting of feature dimensions. Indeed, on closer examination, both the key and the light location are represented by the *same* spatial feature dimension (i.e., left–right). Therefore one could argue that not feature dimensions, rather the respective *sensory* *dimensions* are selectively enhanced by top-down task influences. In other words, the task instruction determines whether a participant attends to either the (visual) light locations or the (haptic) key locations. Subsequently, the attended locations get encoded on the single spatial left–right feature dimension. The fact that this same left–right feature dimension is also used to encode the stimulus location forms the basis of the observed SRC effects.

### Aim of the current study

In line with the above interpretation of the results by Hommel (1993), Memelink and Hommel (2005) demonstrated that mere task instruction may *not* be sufficient to affect action coding if the manipulation does not change the task *goal*. The question then arises: what constitutes a task goal? Does one need to attend to different objects in the environment to selectively enhance sensory coding? Or does the intentional weighting principle apply to more abstract feature codes as well? In the present study we assess the influence of task instruction on automatic processes in stimulus–to–response translation at the feature level.

Since our overall goal is an overarching framework of the interaction between perception and action and cognitive control, the aim of the present study was twofold. First, we were interested to see whether task instruction can change how participants encode a particular movement at the feature level. And, second, we were interested to see whether the outcomes can be accounted for by means of a HiTEC simulation of the task – which could clarify computationally a how task instruction modulates the interplay between perception and action.

In the design of the task there are two important criteria to take into account: (1) the experimental set up needs to employ a *single object* and a *single sensory* *dimension* which can be encoded in *two different feature dimensions*, based on the task instruction. In this way, we can rule out the role of purely object based attention; (2) the experimental set up needs to use a task in which two different interpretations of the same ambiguous movement are – to a certain extent and in the eyes of the participant – equally intuitive and applicable to the observed (sensory) effects of the physical movements. Otherwise, if participants can easily recode the variations in these dimensions into a single intuitive dimension, they will do so; the influence of task instruction will then disappear (cf., Memelink and Hommel, 2005).

With these criteria in mind we opted for a relatively natural scenario rather than responding by pressing keys (see Wang et al., 2007; Yamaguchi and Proctor, 2011 for similar approaches). In a natural scenario – we hypothesized – participants would be more strongly compelled to adhere to the action coding specified by the task instruction. In the present study, participants stood on a Wii balance board and were instructed to imagine standing on either a snowboard or a pair of skis. They had to respond to stimuli by leaning sideways. In the ski condition, this lateral movement was presented as moving the skis to the “*left*” or “*right*,” whereas in the snowboard condition, it was presented as moving the snowboard “*backward*” or “*forward*.” In performing the task, participants could draw on their own motor experience if they had any experience with skiing or snowboarding. Participants who had never skied or snowboarded could still form a mental representation of what it means to be skiing or snowboarding, by combining elements from partial or similar experiences (Barsalou, 2008; Taylor and Zwaan, 2009). For example, they could draw on visual experience (e.g., watching snowboarders on TV), and combine this with related motor experience (e.g., surfing or skateboarding).

**Figure 1:**
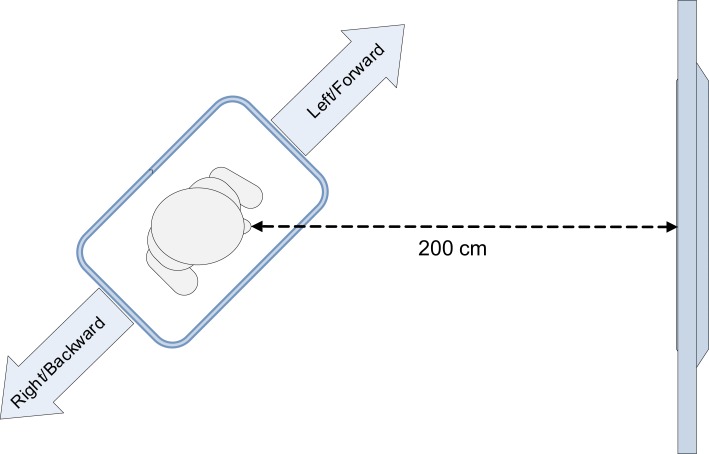
**Setup of the experiment**.

In the experiment, the Wii balance board was oriented diagonally toward the screen displaying the stimuli (Figure 1). The critical stimuli consisted of colored arrows pointing in one of four directions (backward, forward, left, or right). The study used a between-subjects design; participants were either instructed to imagine standing on a pair of skis or on a snowboard, and to respond to the stimulus color by leaning sideways. Given the diagonal orientation of the balance board, the responses simultaneously varied on the left–right dimension and on the forward–backward dimension. We expected that the weighting of the (feature) dimensions would depend on the instruction given to the participant. A skier stands in the same direction as her skis. When she leans to the left or right, this causes the skis to turn into the respective direction. Therefore, participants in the ski condition would encode the lateral leaning movements as “left” and “right.” In contrast, a snowboarder stands on a snowboard perpendicular to its direction of movement. When she leans sideways, the snowboard will slide forward or backward. As a result, we expected that participants in the snowboard condition would not only encode the movements as “left” and “right,” but also as “forward” or “backward.” Therefore, we expected a forward–backward congruency effect to occur in the snowboard condition, but not in the ski condition.

In the next section we describe the methods of the behavioral experiment. We continue with presenting the results, followed by a HiTEC simulation of the study. Finally, we discuss the implications of both our empirical findings and simulation results.

## Material and Methods

### Participants

A total of 83 Dutch undergraduate psychology students from Leiden University (65 women, 18 men) took part in the experiment. In return for their participation they received course credits or a monetary reward of EUR 4.50. Mean age of the participants was 19.8 (SD 2.3).

### Apparatus and stimuli

**Figure 2:**
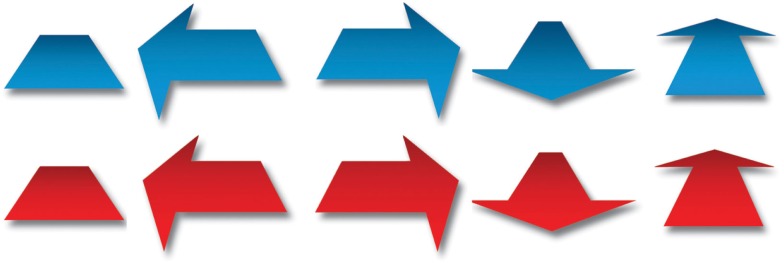
**Experimental stimuli**. Arrows pointing forward, backward, left, right, and direction-neutral stimulus.

The instructions and stimuli were presented on a television monitor with a diameter of 107 cm and a refresh rate of 60 Hz. E-Prime software was used to present the stimuli. Stimuli were blue or red symbols, consisting of one direction-neutral stimulus and arrows pointing in one of four different directions; left, right, forward, or backward (Figure 2). On screen, each stimulus measured approximately 30 cm × 30 cm.

Participants stood on a Wii balance board (51 cm long × 32 cm wide × 5 cm high), which was placed diagonally, at an angle of 45° or −45°, in front of the monitor.

In order to be able to face the monitor, participants who were positioned at the 45° angle always had their left foot forward (i.e., closest to the monitor), and participants at the −45° angle always had their right foot forward. Thus, the participant’s position with respect to the computer screen was determined by the orientation of the balance board.

The distance between the monitor and the center of the balance board was 200 cm (Figure 1). The orientation of the balance board was counterbalanced across participants. Half of the participants stood with their left foot forward, the other half stood with their right foot forward. The participant’s weight distribution on the left–right axis and front–back axis of the balance board was recorded at a frequency of 100 Hz. This was done by custom-made software that polls the sensor values of the balance board, using a Bluetooth connection. To respond to a stimulus, participants had to lean sideways far enough to exceed a predefined threshold on the left–right axis of the balance board. When this threshold was exceeded, the response time and accuracy of the response were logged.

### Procedure

**Figure 3:**
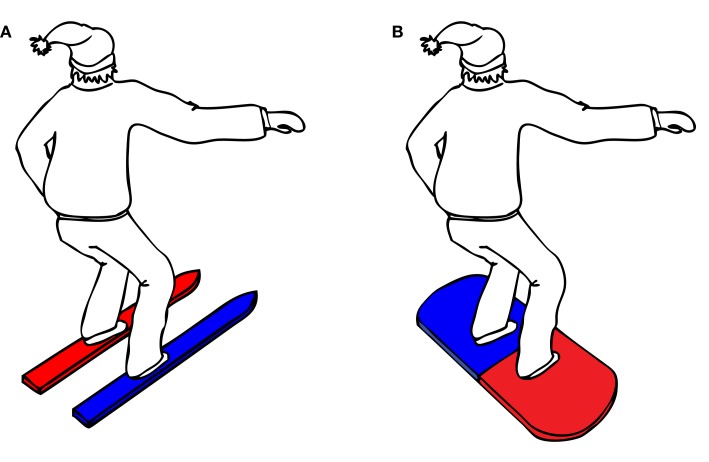
**Illustrations of (A) skier and (B) snowboarder used during instruction**.

The complete experiment lasted approximately 30 min. Upon arrival to the lab, participants were randomly assigned to one of eight counterbalance versions (see Table 1), defined by the instruction (snowboard or ski), the orientation of the balance board (45° or −45°), and the stimulus–response mapping (red–left/blue–right or red–right/blue–left). Participants in the snowboard condition received the following instruction: “*Imagine that you’re standing on a snowboard, which you can move forward or backward by leaning on your front or back leg*,” whereas participants in the ski condition received the alternative instruction: “*Imagine that you’re standing on skis, which you can move to the left or right by leaning on your left or right leg*.” To enhance the context of the task, an illustration of a skier, or a snowboarder was presented, standing in the same position as the participant on the balance board (see Figure 3).

**Table 1:** **Overview of the eight different counterbalance versions of the experiment**.

Task	Position	Instruction
Ski	45° (Left foot forward)	If the image is blue, lean to the left		If the image is red, lean to the right
		If the image is blue, lean to the right
		If the image is red, lean to the left
	−45°(Right foot forward)	If the image is blue, lean to the left		If the image is red, lean to the right
		If the image is blue, lean to the right
		If the image is red, lean to the left
Snowboard	45° (Left foot forward)	If the image is blue, lean forward		If the image is red, lean backward
		If the image is blue, lean backward
		If the image is red, lean forward
	−45°(Right foot forward)	If the image is blue, lean forward		If the image is red, lean backward
		If the image is blue, lean backward
		If the image is red, lean forward

The instruction was followed by a practice block, which contained 24 trials. Each practice trial started with the presentation of the sentence “*Take the start position*” for 1000 ms. Next, the instruction to lean into a particular direction [e.g., “*Move the skis to the left (left leg)*” or “*Move the snowboard forward (front leg)*”] was presented until the participant responded by leaning into the respective direction. In the snowboard condition, the directions were “*backward*” or “*forward*,” whereas in the ski condition the directions were “*left*” or “*right*.” To enhance the encoding of the movements in the appropriate dimension, participants were instructed to mention out loud the direction in which they had to lean. Following a correct response, the word “*correct*” was presented for 1000 ms. Following a response that was incorrect or too slow (more than 5000 ms), the word “*error*” or “*too slow*” was presented for 1000 ms.

After completing the practice trials, participants received the instruction for the experimental trials. They were instructed to respond to the stimulus color by leaning into a particular direction. In the snowboard condition, participants had to respond to red or blue stimuli by leaning forward or backward (e.g., “*If the image is red, lean forward*”). In the ski condition, participants had to respond to red or blue stimuli by leaning to the left or right (e.g., “*If the image is red, lean to the left*”). The actual mapping of color to direction was counterbalanced across participants. In addition, participants were urged to respond as quickly and accurately as possible.

The instruction was supported by the illustration of the skier or snowboarder, in which the two skis or the two sides of the snowboard were colored in the corresponding stimulus color (for example, a skier with a red left ski and a blue right ski, see Figure 3).

Each trial was either neutral (the neutral shape), left–right congruent (left- or right-pointing arrow, corresponding to the horizontal direction of the response), left–right incongruent (left- or right-pointing arrow, opposite to the horizontal direction of the response), forward–backward congruent (forward- or backward-pointing arrow, corresponding to the forward–backward direction of the response), or forward–backward incongruent (forward- or backward-pointing arrow, opposite to the forward–backward direction of the response).

The experiment was divided into four blocks with 50 trials each. Since there were 10 different stimuli (two colors; red and blue, and five orientations; backward, forward, left, right, and neutral), each stimulus was repeated five times during each block. Stimuli were presented in random order. A trial started when the participant had taken the start position and his/her balance was centered on the Wii balance board. After 500 ms, a black fixation cross was presented for 1000 ms, followed by the experimental stimulus. The stimulus remained on the screen until the participant’s response was recorded or until 5000 ms had elapsed. If the response was incorrect or too slow, a feedback screen was presented for 2000 ms, displaying the word “*error*” or “*too slow*.” If the response was correct, no feedback was given. After completing a trial, participants had to return their balance to the center of the balance board. Following each block of 50 trials, there was a short break of 10 s, during which the instruction was repeated. The instruction was visually supported by the same illustration of the snowboarder or skier that had been shown in the initial experimental instruction (Figure 3).

After completing the experimental trials, participants indicated whether they had any experience with skiing or snowboarding. Experienced snowboarders also indicated whether they preferred to snowboard with their left foot forward or their right foot forward.

## Results

The data from eight participants were discarded because they had an overall accuracy level lower than 0.70. For the remaining participants (38 in the Ski condition and 37 in the Snowboard condition) we computed mean reaction times and accuracy for the responses. Incorrect responses (7.8%) were excluded from the reaction time analysis. Furthermore, based on Tukey’s criterion, reaction times below 415 ms and above 1590 ms (5.3%) were also discarded. Mean trimmed reaction times and error rates are presented in Table 2. The reaction times were analyzed with a 2 × 2 × 2 repeated measures ANOVA, with dimension (backward–forward vs. left–right) and congruency (congruent vs. incongruent) as within-subject variables, and instruction (ski vs. snowboard) as between-subject variable.

**Table 2:** **Mean response times (ms) and standard deviations for the different trials in the two instruction conditions**.

Instruction	Dimension	Congruent	Incongruent	Effect (ms)
Ski	Left–right	970 (154.6)	1056 (179.8)	84
	Forward–backward	1006 (159.1)	1011 (169.7)	5
Snowboard	Left–right	922 (134.5)	981 (153.4)	59
	Forward–backward	950 (120.3)	966 (134.0)	16

The majority of participants (27 in the ski group, 18 in the snowboard group) had no experience with snowboarding or skiing, 14 participants had only ski experience (6 in the ski group, 8 in the snowboard group), 5 participants had only snowboard experience (2 in the ski group, 3 in the snowboard group), and 11 participants had both ski and snowboard experience (3 in the ski group, 8 in the snowboard group). Because of the small number of participants in some of the groups, we ignored this factor in the analysis.

There was a main effect of congruency, with congruent trials being faster than incongruent trials, *F*(1,73) = 108.4, *p* < 0.001, $\eta_{p}^{2}=0.60$. In addition, there was a significant interaction between congruency and dimension, *F*(1,73) = 72.5, *p* < 0.001, $\eta_{p}^{2}=0.50$. The congruency effect was larger for the left–right dimension than for the backward–forward dimension. This finding is in line with the left–right prevalence effect found in other studies (e.g., Nicoletti and Umiltà, 1984, 1985; Nicoletti et al., 1988). Different accounts are given for this effect (see e.g., Hommel, 1996; Proctor et al., 2003; Rubichi et al., 2005). We will turn to this matter in the discussion section. Most interestingly, there was a significant three-way interaction between congruency, dimension, and task instruction, *F*(1,73) = 7.1, *p* = 0.01, $\eta_{p}^{2}=0.09$. On the left–right dimension, the congruency effect was significantly larger in the ski condition than in the snowboard condition, *F*(1,73) = 4.5, *p* = 0.04, $\eta_{p}^{2}=0.60$. The opposite result appeared on the front–back dimension; there was a significant congruency effect in the snowboard condition, *t*(36) = 2.4, *p* = 0.02, but not in the ski condition, *t*(37) = 1.0, *p* = 0.33. Although responses in the snowboard condition appeared to be faster in the snowboard condition than in the ski condition, there was no significant main effect of task, *F*(1,73) = 2.7, *p* = 0.11, $\eta_{p}^{2}=0.03$, because the between-subject differences were quite large.

Concluding, significant spatial congruency effects were found both in the left–right dimension and in the forward–backward dimension. Although the instructions did not cause a complete switch of the congruency effects, they modulated the relative size of the effects. On the left–right dimension, the effect was significantly larger in the ski condition than in the snowboard condition. On the forward–backward dimension, the effect was larger in the snowboard condition than in the ski condition. These results suggest that participants in the ski condition may have encoded the movements predominantly as “left” and “right,” whereas participants in the snowboard condition may have encoded the movements also as “forward” and “backward.” Before discussing our results in more detail, we will first present the HiTEC model and explain how this model can account for our findings.

## HiTEC Simulation

The experiment was simulated using the HiTEC connectionist model (Haazebroek et al., submitted) in order to explain the results presented above. More specifically, we aimed to simulate the way in which the task context modulates the interaction between stimulus perception and response planning. HiTEC is being developed to computationally specify the mechanisms proposed in TEC (Hommel et al., 2001) in terms of neurally plausible representations and connections. It is the aim to validate TEC’s principles and assumptions by means of simulations of particular empirical studies using specific instances of HiTEC (Haazebroek et al., 2009, 2010). In this section we first describe the basic principles of connectionist modeling and discuss global cortical connectivity. We then proceed to discuss HiTEC’s general structure and relate this to TECs main assumptions. Finally, we discuss the specific simulation set up for the current study, the simulation results, and the model dynamics in order to account for the empirical findings from Section “Results.”

### Connectionist modeling and cortical connectivity

**Figure 4:**
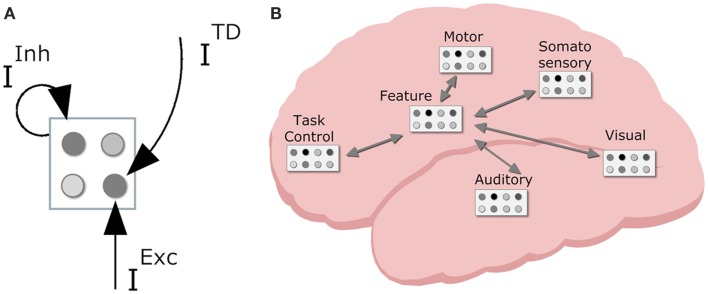
**Neurodynamical modeling approach, with (A) cortical map with neuron populations with various inputs (TD, top down; Inh, lateral inhibition; Exc, excitatory input); (B) tentative locations of various cortical maps in the primate brain with sensory maps in sensory regions, task control maps in the frontal lobe, motor maps in motor area, and intermediate feature maps mediating between lower and higher region maps**.

In order to devise a neurally plausible model, it is important to consider both representations and patterns of connectivity in the brain. Regarding the former, the primate cortex is composed of a vast amount of spiking neuron cells. The local interactions between these neurons are largely random, but on a group level – a neuron population – the global population activity (i.e., mean spike frequency) can be considered deterministic (Wilson and Cowan, 1972). That is, mean activation depends on various inputs and the decay of the neuron population (see Figure 4A for a visual illustration of neuron populations and their inputs).

As we consider a neuron population the basic unit, we can model these neurodynamics with an interactive activation connectionist network (Rumelhart et al., 1986) of units and connections. The propagation of activation of a unit is described by the following equation:

(1) $$\begin{array}{llll} A_{i}\left(t+1\right) & =\left(1-d_{\text{a}}\right)\times \;A_{i}\left(t\right)+\left(1-A_{i}\left(t\right)\right) & & \\ & \;\times \left(\text{Ex}{\text{c}}_{i}+\text{T}{\text{D}}_{i}+\text{Nois}{\text{e}}_{i}\right)+\text{In}{\text{h}}_{i}\times A_{i}\left(t\right) \end{array}$$

This equation states that the activation of unit *i* is determined by its current value, a decay rate *d*_a_ (default value of 0.1 in current simulations), excitatory input Exc*_i_*, top-down input Td*_i_*, lateral inhibitory input Inh*_i_*, and background noise input Noise*_i_* (standard Gaussian random additive noise with mean: 0.025, and SD 0.015) The excitatory input is either external stimulation (0.6 in current simulations) or excitatory input originating from connected feedforward units, which is computed according to the following equation:

(2) $$\text{Ex}{\text{c}}_{i}=\underset{k}{\sum }w_{k}^{+}F\left(A_{k}\left(t\right)\right)$$

This equation states that the excitatory input consists of the weighted sum of the outputs of all connected feedforward units. Here, *w*^+^ are the positive weights of the connections from unit *k* to unit *i*. The output of a unit is a non-linear function of its activation value using the following function with parameters na (4.0 in current simulations) and qa (0.9 in current simulations).

(3) $$F\left(A_{i}\right)=\frac{A_{i}^{\text{na}}}{{\left(\text{qa}\right)}^{\text{na}}+A_{i}^{\text{na}}}$$

Top-down input to a unit originates from units “later” or “higher” in the processing flow and are considered to only enhance activation. This is realized by means of the following computation of top-down input:

(4) $$\text{T}{\text{D}}_{i}=\underset{k}{\sum }w_{k}^{+}F\left(A_{k}\left(t\right)\right)\times \frac{\max\left(A_{i}\left(t\right)\;\times \left(1-d_{a}\right)-\text{VT},0\right)}{1-\text{VT}}$$

Here, *d*_a_ is the same activation decay rate (0.1) as in Eq. 1 and VT (0.5 in current simulations) is a voltage threshold (see also Tononi et al., 1992). When unit *i* has an activation level higher than this threshold, top-down input from connected units is taken into account and rescaled in proportion to the voltage threshold. Conversely, if the unit’s scaled activation level is lower than the voltage threshold, this input is discarded.

Finally, inhibition is computed using paired inhibitory units (see also Deco et al., 2002). Each unit has a paired inhibitory unit that receives excitation from the (excitatory) unit and sends inhibition (through negative weights) to (excitatory) units within the same map (i.e., lateral inhibition). This is computed using the following equation:

(5) $$\text{In}{\text{h}}_{i}=\underset{k}{\sum }w_{k}^{-}F\left(A_{k}\left(t\right)\right)$$

Here, *k* denotes the inhibitory paired unit belonging to any other unit than unit *i* in the map and *w*^−^ are the negative connection weights (−0.75 in current simulations). The activation of inhibitory units is updated in a similar fashion as the excitatory units, but their input can only be excitatory and originating from the paired excitatory units. Note that we do not depict inhibitory units in any model diagram for clarity reasons and that by “code” we always refer to the excitatory unit. In our current simulations the connection weight from an excitatory unit to its paired negative unit is 1.25.

Weights between units are considered to be able to change over time as a result of learning. The weight change depends on the level of activation of both units during learning following Hebbian learning. Weight (bound to vary between 0.0 and 1.0) learning is governed by the following equation:

(6) $$\begin{array}{llll} w_{jk}\left(t+1\right) & =\left(1-d_{w}\right)\times w_{jk}\left(t\right)+\text{LR}\;\times A_{j}\left(t\right)\;\times A_{k}\left(t\right) & & \\ & \;\times \left(1-w_{jk}\left(t\right)\right) \end{array}$$

In these equations, *w_jk_* is the weight from unit *j* to unit *k*, the *d_w_* weight decay rate (0.0005 in current simulations) ensures that only repeated co-activations result in stable weight learning, LR (0.1 in current simulations) denotes the learning rate (i.e., the magnitude of the change in weights for each learning trial), *A_j_*(*t*) is a value based on the activation of feature code unit *j*, *A_k_*(*t*) is a value based on the activation of motor code unit *k*.

In sum, these modeling equations and parameters allow for a biologically plausible simulation of activation propagation through a network of units. Higher decay rates make units decay faster; lower decay rates keep units very active for a longer period of time. Higher input values for external input and stronger weights between units result in faster activation propagation. Higher voltage thresholds make unit activation to a lesser extent enhanced by top-down input; conversely, lower voltage thresholds lead to earlier and stronger influence of top-down modulation on unit activation. Stronger weights between excitatory and inhibitory units strengthen the lateral inhibition mechanism. As a result, they reduce the time required to settle the competition between the units within a shared map, after which only one unit remains strongly activated. Lower weights, conversely, lengthen this time to convergence.

With this basic connectionist machinery in place we can turn to (global) cortical patterns of connectivity. The neurons in primate cortex are organized in numerous interconnected cortical maps (see Figure 4B). This allows the brain to encode perceived objects in a distributed fashion. That is, different features are processed and represented across different cortical maps (e.g., DeYoe and Van Essen, 1988), coding for different perceptual modalities (e.g., visual, auditory, tactile, proprioceptive), and different dimensions within each modality (e.g., visual color and shape, auditory locationn, and pitch). Each sensory cortical map contains neurons that are responsive to specific sensory features (e.g., a specific color or a specific visual location). Sensory representations are known to have stronger decay than higher level representations; in simulations, this is typically reflected by a stronger decay rate (0.2 in current simulations) for sensory code units than for other units (0.1 default decay rate). Cortical maps in the motor cortex contain neurons that code for more or less specific movements (e.g., the muscle contractions that produce the movement of the hand pressing a certain key, or more complex movement such as shifting one’s weight to the right). Higher up in the processing stream there are cortical maps containing neurons that are receptive to stimulation from different modalities. In effect, they are considered to integrate information from different senses and modalities. Finally, neurons in the prefrontal cortex are involved in task-generic cognitive control (Duncan and Owen, 2000). These levels of representation form the basis of the HiTEC model.

### HiTEC model

**Figure 5:**
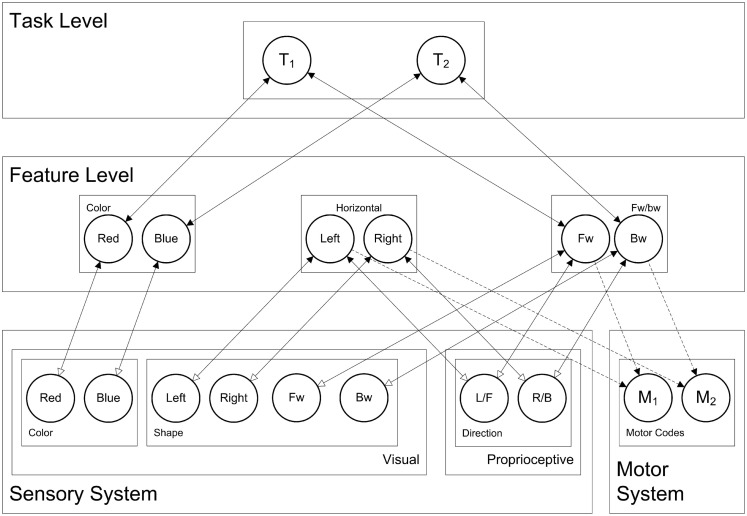
**HiTEC model of the balance board task**. Solid lines depict fixed connections, dashed lines are connections that are learned during action–effect learning. Depicted is the model in snowboard instruction condition, where the left leg is the front leg, and where a red stimulus requires a forward response (and a blue stimulus a backward response). Note that “forward” and “backward” feature codes are abbreviated as “FW” and “BW” and that “L/F” denotes the ambiguous left/forward sensory code and “R/B” the right/backward sensory code.

Now, taking this general cortical layering, connectivity, and dynamics, the question arises: how are these connectionist network units interconnected in order to yield behavior that is typically associated with processes like stimulus perception, response selection, and response planning? To this end, we present the HiTEC connectionist model, based on TEC’s main assumptions. HiTEC’s general structure contains sensory maps, feature maps, a task map, and a motor map, as depicted in Figure 5. Each map resembles a cortical map and contains codes implemented as connectionist network units as described above.

Note that Figure 5 shows only those sensory maps that are relevant for modeling the current experiment: visual color, visual shape, and proprioceptive direction. However, other specific instances of the model may include other sensory maps as well (e.g., auditory maps). Although motor codes could also be organized in multiple maps, in the present version of HiTEC, we consider only one basic motor map with a set of motor codes.

Theory of event coding’s notion of feature codes (Hommel et al., 2001) is captured at the feature level by codes that are connected to and thus grounded in both sensory codes and motor codes. Crucially, the same (distal) feature code (e.g., “left”) can be connected to multiple sensory codes (e.g., “left proprioceptive direction” and “left visual shape”). Thus, information from different sensory modalities and dimensions is combined in one feature code representation. It is assumed that feature codes arise from regularities in sensorimotor experience, presumably by detecting co-occurrences of sensory features. The distal feature “left,” for example, could arise from perceptual experience of numerous objects that were visible and audible on the left. Future encounters of objects audible on the left activate the “left” feature code which – by means of its connections to both “left auditory location” and “left visual location” – will enhance the processing of visual left locations. In other words, hearing something on the left will result in expecting to see something on the left as well, which seems to be quite useful, for example when visual sensory input is degraded. In the present HiTEC model, for current simulation purposes, we assume that the feature codes (and their connections to sensory codes) already exist.

Finally, the task level contains generic task control codes that reflect alternative stimulus–response combinations resulting from the task context. Different task codes reflect different response choice options within the task context (i.e., the typical “*if X then do Y*” task rules). Task codes connect to feature codes only, both the feature codes that represent stimuli and the feature codes that represent responses, in close correspondence with the current task context. For the current study the appropriate task codes, feature codes, and their connections are depicted in Figure 5 (i.e., snowboard condition).

In line with TEC, responses are encoded in terms of their perceivable effects. This assumption is derived from the ideomotor theory (Hommel, 2010, 2013), which presumes that when an action is executed, the motor pattern is automatically associated to the perceptual input representing the effects of the action in the distal environment. For example, a novice snowboarder learns that by shifting her weight laterally, she can control the forward movement of her snowboard. She may learn that her snowboard slides forward when she leans to the left, and that it slides backward when she leans to the right (the precise mapping depends on the snowboarder’s position on her board). Thus, when she leans to the left and moves forward as a result, the action is not only perceived and represented as “left,” but also as “forward.” After learning these action–effect associations, the snowboarder can plan and control her movements by anticipating their perceptual effects; that is: (re-)activating the motor patterns by intentionally (re-)activating the associated feature codes. Thus, when an expert snowboarder intends to move “forward,” she will automatically shift her weight into the appropriate direction.

Note that the basic dynamics of connectionist modeling used in HiTEC resembles those used in typical connectionist network models (PDP models, e.g., Rumelhart et al., 1986). However, here, input from feedforward and feedback connections is combined, resulting in activation flowing back and forth between units on various levels of coding. This sets the type of modeling apart from – for example – various feedforward PDP models of automaticity (e.g., Cohen et al., 1990; Zorzi and Umiltà, 1995). In addition, codes within the same map inhibit each other. Together, this results in a global competition mechanism in which *all* codes participate, from the first processing cycle to the last.

### Simulating behavioral studies

Using HiTEC, specific behavioral studies can be simulated. In behavioral studies, participants typically perceive a stimulus and select and plan an action response. In general, a stimulus is presented to the HiTEC model by applying excitatory input to its sensory codes. After a number of cycles of internal processing a motor code becomes highly activated. When this motor code activation exceeds the set response threshold, this response is considered to be produced. Codes and their connections reflect both prior experience and task instructions. By measuring the number of cycles necessary to produce a motor response in various conditions, reaction time can be computed and compared to human data. More importantly, however, the internal dynamics of the model can shed light on the computational principles underlying both the simulation and the empirical results.

In behavioral experiments, participants typically receive a verbal instruction of the task. In HiTEC, a verbal task instruction is internalized as connections between feature codes (cf., in humans presumably using verbal labels, Hommel and Elsner, 2009) and generic task codes. Due to the mutual inhibitory links between these task codes, they will compete with each other during the task. Currently, the connections between feature codes and task codes are systematically set by hand in close correspondence with the task instruction.

Connections between feature codes and motor codes are explicitly learned, following the general set up of action–effect learning paradigms (e.g., Elsner and Hommel, 2001): at first, a random motor code is activated, comparable to the spontaneous motor babbling behavior of newborns. This leads to a change in the environment (e.g., the left hand suddenly touches an object) that is registered by sensory codes. Activation propagates from sensory codes toward feature codes. Subsequently, associations are learned between the active feature codes and the active motor code using the Hebbian learning equation described in Section “Connectionist Modeling and Cortical Connectivity.” Once associations between motor codes and feature codes exist, they can be used to select and plan actions. Planning an action is realized by activating the feature codes that correspond to its perceptual effects and by propagating their activation toward the associated motor codes. Initially, multiple motor codes may become active as they typically fan out associations to multiple feature codes. However, some motor codes will have more associated features and some of the associations between motor codes and feature codes may be stronger than others resulting in variations in dynamics. In time, the network will converge toward a state where only one motor code is strongly activated, which leads to the selection of that motor action.

When a stimulus in an experimental trial is presented, the corresponding sensory codes are activated. Activation gradually propagates toward the associated feature codes and toward those task codes that were associated during task preparation. Consequently, activation is propagated to feature codes that correspond to (perceptual effects of) responses and finally toward motor codes (that were associated during action–effect learning).

Note that all codes are involved from stimulus onset and gradually activate each other; as a result competition takes place between feature codes, between task codes, and between motor codes, simultaneously. Once any one of the motor codes is activated strongly enough, it leads to the execution of the respective motor response to the presented stimulus. In our simulations, this marks the end of a trial.

In general, the passing of activation between codes along their connections is iterated for a number of cycles, which allows for the simulation of reaction time (i.e., number of cycles from stimulus onset to response selection) until the activation level of any one of the motor code reaches a set threshold value (0.6 in current simulations).

### Modeling the current empirical study

The current study involves colored arrow-shaped stimuli and responses that require a participant to move his/her balance to a certain direction (left/forward and right/backward). In order to be able to register these sensations, the HiTEC model is equipped with sensory maps for color, shape, and proprioceptive direction. In addition, two movements are included in the motor map. We could have included more sensory maps or motor codes, but these would not be activated by any stimulus in the current study. For clarity reasons, we restricted the model to relevant codes only.

The task context includes instructions for responding to the stimulus color (“*red*” or “*blue*”), by moving either “*left*” vs. “*right*” or “*forward*” vs. “*backward*,” depending on the instruction group. We have included feature codes for these terms and have connected these codes to task codes appropriately. For each simulated subject, there are only two task rules to choose from, reflected by the two task codes in the task map. Figure 5 depicts the codes and connectivity for a simulated subject in the snowboard condition who was instructed to respond to red stimuli by moving forward, and to blue stimuli by moving backward, as can be seen by the connections between feature codes and task codes.

As illustrated in Figure 5, sensory codes are connected to feature codes (feedforward weight 0.4, feedback weight 3.0). Stimulus related feature codes are connected to task codes (feedforward weight 1.5, feedback weight 0.2) and task codes to response related feature codes (feedforward weight 1.5, feedback weight 0.2) allowing activation to propagate from sensory codes to stimulus related feature codes to task codes to response related feature codes. Connections between feature codes and motor codes are explicitly learned. Importantly, in the current simulation, we have taken into account that the cognitive system has more experience with coding for “left” and “right” than is the case for “forward” and “backward.” In the model this is realized by setting the weights from sensory codes toward “forward” and “backward” slightly lower (0.3 rather than 0.4).

Note that the sensory codes for proprioceptive direction (i.e., proprioceptive map in Figure 5) are not considered “left” vs. “right” or “forward” vs. “backward” by themselves. They represent two ambiguous sensations that can activate feature codes in both feature dimensions. We shall see that task context (i.e., the connections between feature codes and task codes, in close correspondence with the task instruction) determines to what extent this sensation is perceived as “left” vs. “right” or “forward” vs. “backward.”

The HiTEC simulation of the current empirical study consists of 40 simulated subjects in the ski condition and 40 simulated subjects in the snowboard condition. For each simulated subject, first the instruction is internalized by setting its task code–feature code connections appropriately; then, during 20 training trials feature code–motor code connections are learned, and finally, 20 repetitions of the 10 experimental trials (i.e., 2 colors × 5 shapes) are performed. This corresponds to the design of the empirical study as discussed in Section “Material and Methods.” Each individual simulated subject has its own random noise resulting in subtle individual differences in processing and in variance in behavior (i.e., varying reaction times) as is the case with individual human participants.

### Simulation results

**Figure 6:**
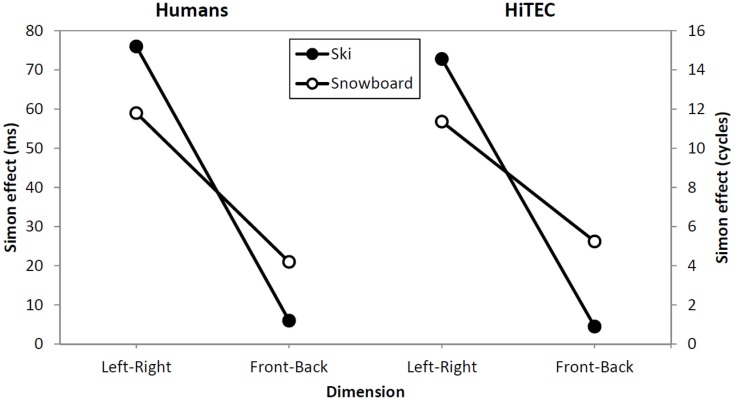
**Comparison between human data (left) and simulation results (right)**. Lines depict the effect sizes for both instruction groups (ski and snowboard) and both congruency dimensions (left–right and forward–backward).

Table 3 shows the average number of cycles from stimulus onset until response selection for both instruction conditions and both congruency levels. As accuracy was 1.0 for all simulated subjects, it was not regarded in the analysis. The three-way interaction between congruency, dimension, and task instruction found in the experiment was replicated in the simulation, as depicted in Figure 6. The left–right congruency effect was larger in the ski condition, whereas the forward–backward congruency effect was larger in the ski condition. We now explain how these results arose in the simulation by discussing the model dynamics in more detail.

**Table 3:** **Average number of processing cycles from stimulus onset until stimulus selection in the HiTEC model, based on all 80 simulated subjects**.

Instruction	Dimension	Congruent	Incongruent	Effect
Ski	Left–right	14.7	29.2	14
	Forward–backward	17.1	18.0	0.9
Snowboard	Left–right	15.6	27.0	11.4
	Forward–backward	15.7	21.0	5.3

Not that the HiTEC simulation only covers a part of the entire process of stimulus to response production in humans. The actual movements, for example, are included in the empirical reaction times (Table 2) but are not part of the simulation reaction times (Table 3). This results in larger relative effect sizes in the simulation results as compared to the empirical data.

### Model dynamics during simulation

Although the stimuli and responses are equal for both instruction groups, the congruency effects differ. These differences between the groups are the result of several dynamics of the model, as we will now explain.

The task instruction is reflected by connections between task codes and feature codes. These connections are bidirectional. As a consequence, activating a feature code will activate each connected task code, which on its turn will activate or enhance all connected feature codes, including the feature code that activated the task code in the first place (i.e., recurrent connectivity). This means that the mere fact of being connected to a task code will further enhance the activation of a feature code. For the ski instruction group, this means that “left” and “right” feature codes receive this enhancement, for the snowboard group this is the case for the “forward” and “backward” feature codes.

**Figure 7:**
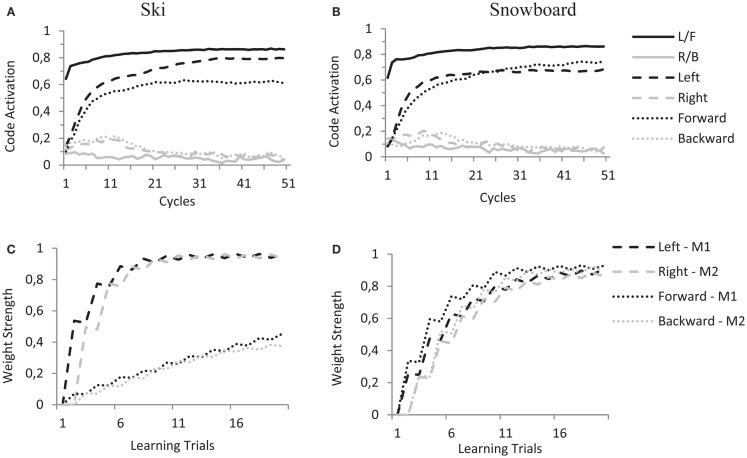
**HiTEC simulation graphs of one simulated subject in the ski condition (A,C) and one simulated subject in the snowboard condition (B,D) during learning trials**. **(A,B)** Show code activations resulting from the perception of the ambiguous action–effect (balance toward “left”/“forward”). Due to differences in task code–feature code wiring there is difference in recurrency and therefore slight differences in code activation (“left” vs. “forward”) in the two instruction conditions. In the **(C,D)**, that show the weight strength of a selection of feature code–motor code connections during all learning trials, it is clear that during the learning trials this difference in code activation accumulates to a substantial difference in the learned action–effect weights.

Crucially, this selective enhancement is already at play during the learning trials. When a motor code is activated during a learning trial, and its effects are presented to the model, the mere connections between feature codes and task codes will enhance either the “Left” and “Right” feature codes (in the ski condition) or the “Forward” and “Backward” feature codes (in the snowboard condition) and thereby determine the coding of the ambiguous sensation. When the action–effect produced by “M1” is presented (i.e., activating the “L/F” proprioceptive code) this results in a slightly higher activation for the “Left” feature code in the ski condition and a slightly higher activation for the “Forward” feature code in the snowboard condition, as shown in Figures 7A,B. When the action–effect produced by “M2” is presented (i.e., activating the “R/B” proprioceptive code), this works in similar fashion.

During the 20 learning trials, this minimal difference in feature code activation results in pronounced differences in the weights learned (see Figures 7C,D) and prepares the model for the experimental trials. Note that in the ski condition, the weights between the “Left”/“Right” feature codes and motor codes are strong and the weights between the “Forward”/“Backward” feature codes and motor codes are rather moderate (Figure 7C). This is due to both the connections between the task codes and the “Left”/“Right” feature codes and the stronger connections between sensory codes and the “Left”/“Right” feature codes (as compared to the connections between sensory codes and the “Forward”/“Backward” feature codes). In the snowboard condition, the weights between the “Left”/“Right” feature codes and the motor codes are roughly equally strong as the weights between the “Forward”/“Backward” feature codes and the motor codes (Figure 7D). This is due to the “Forward”/“Backward” feature codes being connected to the task codes, resulting in top-down enhancement of these feature codes. At the same time, the “Left”/“Right” feature codes receive more excitatory input due to their stronger connections with the sensory codes.

During the subsequent experimental trials, the model is set to respond to stimulus color and automatically takes stimulus direction into account (stimulus–response congruency, SRC). This is a result from the fact that the model codes for responses and stimuli using common spatial feature codes. In the ski condition, the feature codes “Left” and “Right” are used to encode the responses. When perceiving a horizontal arrow stimulus, however, “Left” and “Right” are also used to encode this stimulus. When a congruent stimulus is presented, the corresponding feature code is already activated to encode this stimulus and therefore speeds up the encoding of the response. When an incongruent stimulus is shown, the wrong feature code is activated which slows down the activation – by means of lateral inhibition – of the correct response feature. This results in longer reaction times for incongruent than for congruent stimuli.

**Figure 8:**
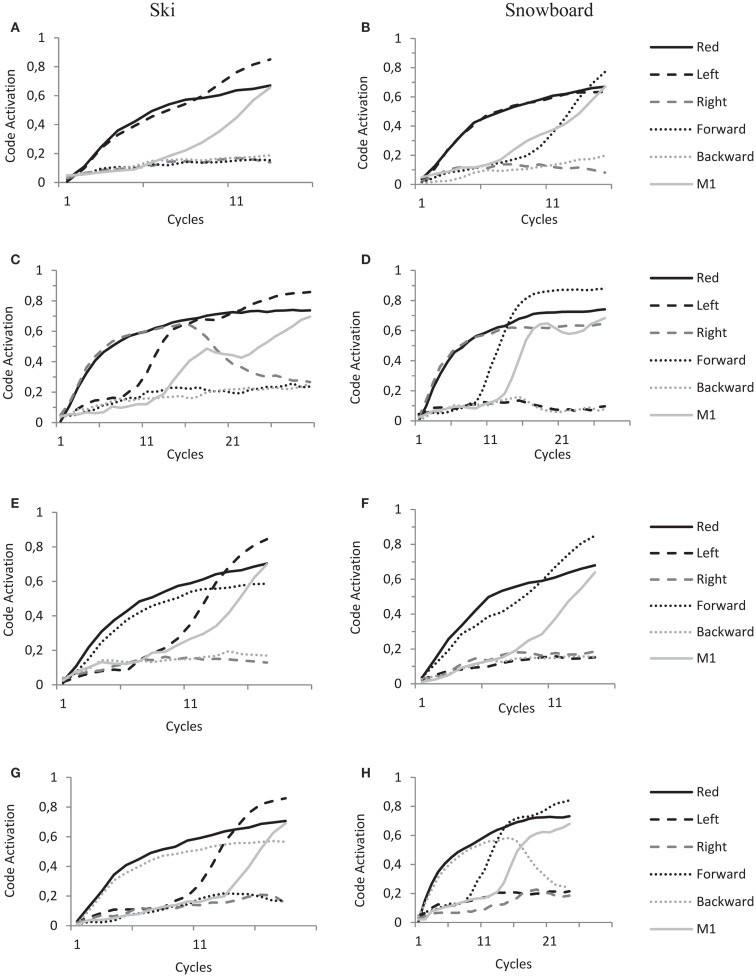
**HiTEC Simulation graphs of ski condition (A,C,E,G) and snowboard condition (B,D,F,H) during the experimental trials**. All panels show code activations of some of the feature codes and a motor code (M1) during the cycles of a single trial. Solid black lines denote the activation of the “Red” feature code, solid gray lines denote the activation of the “M1” motor code, dashed lined the activation of the “Left” and “Right” feature codes and dotted lines the activation of “Forward” and “Backward” feature codes. Trials start with stimulus presentations, hence the fast increase of feature codes that are connected to the sensory codes activated by stimulus presentation. Trials end when a motor code (in these trials motor code M1) reaches the response threshold of 0.6. See text for further explanations of the dynamics leading to action selection.

Now, the overlap between feature codes of stimulus and response obviously depends on the spatial coding of the response. As a result of task instruction and subsequent action–effect learning, this is different for the ski group and snowboard group. We now describe in detail the dynamics of the model during the experimental trials in both ski and snowboard conditions and for each type of stimulus (left–right congruent and incongruent, forward–backward congruent and incongruent) as depicted in the panels of Figure 8.

In panel A, a red left arrow stimulus is presented to the model in the ski condition, resulting in an initial increase of activation of “Red” and “Left” feature codes. In line with the ski task set, activation propagates from “Red” to a task code and to the “Left” feature code. This overlap results in a fast increase of activation of the “Left” feature code. In the ski condition the “Left” feature code is strongly connected to “M1,” resulting in fast activation propagation toward motorcode “M1” and fast action selection. This explains the relatively shorter reaction times for the left–right congruent trials in the ski condition.

In panel B, a red left arrow stimulus is presented to the model in the snowboard condition, resulting in an initial increase of activation of “Red” and “Left” feature codes. In line with the snowboard task set, activation propagates from “Red” to a task code and to the “Forward” feature code; hence the subsequent increase in activation of the “Forward” feature code. In the snowboard condition, “Left,” “Right” *and* “Forward” and “Backward” feature codes are strongly connected to the motor codes (as depicted in Figure 7B). Thus, both “Left” and “Forward” now propagate activation toward motor code “M1” resulting in fast action selection. This explains the relatively shorter reaction times for the left–right congruent stimulus trials in the snowboard condition.

In panel C, a red right arrow is presented to the model in the ski condition, resulting in initial increase of activation of “Red” and “Right” feature codes. In line with the ski task set, activation propagates from “Red” to a task code and to the “Left” feature code; hence the subsequent increase in activation of the “Left” feature code. Now, both “Left” and “Right” feature codes are active and highly competing. They are both strongly connected to different motor codes that both receive activation and also compete with each other. This competition takes time and lengthens the trial.

In panel D, a red right arrow is presented to the model in the snowboard condition, resulting in initial increase of activation of “Red” and “Right” feature codes. In line with the snowboard task set, activation propagates from “Red” to a task code and to the “Forward” feature code, hence the subsequent increase in activation of the “Forward” feature code. Now, the “Forward” feature code is strongly connected to the M1 motor code, the motor code to be selected. The “Right” feature code, however, is (even more) strongly connected to the “M2” motor code. As both “Forward” and “Right” feature codes are highly activated and propagate activation to both motor codes, it takes longer for the system to settle this competition. This explains the relatively longer reaction times for the left–right incongruent stimulus trials in the snowboard condition.

In panel E, a red forward arrow is presented to the model in the ski condition, resulting in an initial increase of activation of “Red” and “Forward” feature codes. In line with the ski task set, activation propagates from “Red” to a task code and to the “Left” feature code; hence the subsequent increase in activation of the “Left” feature code. Now, in the ski condition the “Left” feature code is strongly connected to the “M1” motor code, the motor code to be selected. The “Forward” feature code, however, is very weakly connected to the “M1” motor code. Thus the activation mainly propagates from the “Left” feature code toward the “M1” motor code resulting in a speedy selection of the “M1” motor code, whereas the activation of the “Forward” feature code has minimal influence. This explains the unaffected reaction times for the forward–backward congruent stimulus trials in the snowboard condition.

In panel F, a red forward arrow is presented to the model in the snowboard condition, resulting in an initial increase of activation of “Red” and “Forward” feature codes. In line with the snowboard task set, activation propagates from “Red” to a task code and to the “Forward” feature code. This overlap results in fast increase of “Forward” feature code activation. In the snowboard condition the “Forward” feature code is strongly connected to “M1,” resulting in fast activation propagation toward “M1” and fast action selection. This explains the relatively shorter reaction times for the forward–backward congruent trials in the snowboard condition.

In panel G, a red backward arrow is presented to the model in the ski condition, resulting in an initial increase of activation of “Red” and “Backward” feature codes. In line with the ski task set, activation propagates from “Red” to a task code and to the “Left” feature code, hence the subsequent increase in activation of the “Left” feature code. Now, in the ski condition the “Left” feature code is strongly connected to the “M1” motor code, the motor code to be selected. The “Backward” feature code is connected to the “M2” motor code, introducing competition. However, in the ski condition this latter connection is very weak. Thus the activation mainly propagates from the “Left” feature code toward the “M1” motor code resulting in a speedy selection of the “M1” motor code, whereas the activation of the “Backward” feature code has minimal influence. This explains the unaffected reaction times for the forward–backward incongruent stimulus trials in the snowboard condition.

In panel H, a red backward arrow is presented to the model in the snowboard condition, resulting in an initial increase of activation of “Red” and “Backward” feature codes. In line with the snowboard task set, activation propagates from “Red” to a task code and to the “Forward” feature code. Now, both “Forward” and “Backward” feature codes are active and highly competing. They are both strongly connected to different motor codes that also compete. This competition takes time and lengthens the trial, explaining the relatively longer reaction times for the forward–backward incongruent stimulus trials in the snowboard condition.

In sum, the stronger connections between sensory codes and the “Left”/“Right” feature codes (as compared to the weaker connections between sensory codes and the “Forward”/“Backward” feature codes) together with the differences in mere connectivity between feature codes and task codes – which results from different task instructions – yield a pattern of left–right and forward–backward SRC effects that is comparable to the findings from the empirical study.

## Discussion

The Simon effect is known as a particularly robust effect. The empirical study presented here uses a two-dimensional Simon task with two groups of participants who only differ in the instruction (i.e., ski vs. snowboard) they received. And yet, the presence and size of the Simon effect is strongly dependent on the instruction: the left–right congruency effect is larger in the ski condition than in the snowboard condition, while the forward–backward effect only appears in the snowboard condition. Obviously, then, the task instruction moderates the internal translation process from stimulus to response.

Using the TEC, these results could be explained in terms of feature code overlap and intentional weighting: the task context modulates to what extent a feature dimension (i.e., forward–backward or left–right) is used for response coding. Since these feature codes are used both for stimulus encoding and response planning, this results in either facilitation or interference, yielding a stimulus–response congruency (SRC) effect. Simulations using the HiTEC model show how this result may emerge. Task instruction is implemented as connections between feature codes and task codes, closely following the verbal instructions. This mere connectivity automatically results in specific recurrency that selectively enhances either the “Left” vs. “Right” or the “Forward” vs. “Backward” feature codes when perceiving action–effects. This leads to differences in action–effect weight learning and subsequently in how a response is encoded. These differences in response coding, in turn, influence the degree in which the feature codes representing stimuli and responses overlap, giving rise to different SRC effects across conditions.

The data from the empirical study and the results from the simulation clearly show a stronger congruency effect for the left–right dimension than for the forward–backward dimension (see Figure 6, depicted effect sizes are listed in Tables 2 and 3). As mentioned in Section “Results,” the asymmetry in the empirical data is in line with the left–right prevalence effect found in other studies (e.g., Nicoletti and Umiltà, 1984, 1985; Nicoletti et al., 1988). In the current study, we hypothesize that the use of left and right feet – for both left–right and forward–backward responses – may have yielded this prevalence effect (cf. Hommel, 1996). In more general terms, it could be argued (Rubichi et al., 2005) that the right–left discrimination is over-learned and produces faster processing than discriminations on other dimensions. In the model, the left–right dimension was enhanced by strengthening the connection between the sensory codes and feature codes (0.4 for connections to “Left”/“Right,” 0.3 connections to for “Forward”/“Backward”). This resulted in a left–right prevalence effect, similar to the effect found in the empirical data.

### Related work

Our findings are in line with earlier work on the impact of instructions (Hommel, 1993) and otherwise induced task-relevance of stimulus and response dimensions (Memelink and Hommel, 2005) on the Simon effect. Indeed, the effect of task goals on the interaction between perception and action in this study can be ascribed to the basic principle of intentional weighting (Memelink and Hommel, 2012). It should be noted, however, that although the current study shows strong resemblance to the experiment conducted by Hommel (1993), the studies differ in *how* intentional weighting is assumed to be at play. In Hommel (1993), different aspects of the action–effect (i.e., light vs. key) contributed selectively to the *same feature dimension* (i.e., left–right) depending on the task instruction. Describing that task in terms of ”*key pressing*” focused the (spatial) attention on the keys and increased the contribution of key location to the left–right dimension, whereas describing it in terms of ”*light switching*” focused attention on the lights and increased the contribution of light location to the left–right dimension. Subsequently, the stimuli were encoded using this same left–right dimension. This resulted in either facilitation or interference yielding the observed SRC effect. This is fully in line with HiTEC logic, and it has been successfully replicated in HiTEC (Haazebroek et al., submitted).

In contrast, in the current study, a *single sensory dimension* (i.e., proprioceptive balance) was assumed to map onto *two distinct feature dimensions* (i.e., left–right and forward–backward). Here, task instruction modulated the relative weighting of these two feature dimensions in the coding of the response. Subsequently, left vs. right directed stimuli were encoded using the left–right feature dimension and forward vs. backward directed stimuli were encoded using the forward–backward feature dimension. The relative weighting of these feature dimensions – modulated by task instruction – determined the relative sizes of the left–right SRC effects and forward–backward SRC effects, as observed in both the empirical data and simulation results. Indeed, the present empirical study and simulation results demonstrate that intentional weighting is not limited to weighting sensory dimensions, as demonstrated by Hommel (1993) and simulated by Haazebroek et al. (submitted), but also extends to weighting abstract feature dimensions.

Yamaguchi and Proctor (2011) also found that the SRC effect depends on the attentional demands of the task. In their study participants controlled a simulated aircraft. A response yielded action–effects on multiple dimensions: movement of the aircraft, movement of the horizon and the physical joystick movement. In this study, SRC effects depended on whether the (visual) emphasis was on the orientation of the aircraft (i.e., aircraft tilt, fixed horizon) or of the horizon (i.e., fixed aircraft, horizon tilt), which resonates well with our findings. Their work on a multidimensional vector model of SRC (Yamaguchi and Proctor, 2012) also addresses the issue of task context in the Simon task. They mathematically model the S–R vector space and treat stimulus features and response features in similar fashion, which is completely in line with our HiTEC model. HiTEC, however, is not aimed at mathematical minimalism, rather at biological plausibility: connectionist codes with activation dynamics that approximate biological neuron populations, bi-directional connections, and within-layer lateral inhibition.

At first sight, the general architecture of HiTEC, a model of codes, and connections, is in line with existing models (e.g., Zorzi and Umiltà, 1995; Kornblum et al., 1999), but there are some crucial differences to be noted: in HiTEC: (1) responses are coded as motor codes which are associated with feature codes as a result of *learning* rather than as a fixed connotation; (2) compatibility effects arise from the fact that the *same* feature codes are used to represent stimuli and responses at the feature level, rather than assuming spatial similarity between stimuli and responses; (3) in line with the response-discrimination hypothesis (Ansorge and Wühr, 2001), the task instruction determines the response coding and thus influences SRC.

Moreover, the model is compatible with the main claims of embodied cognition theories. In fact, HiTEC’s concepts are entirely grounded in sensorimotor experience and even the grounding process itself is explicitly modeled. In line with TEC (Hommel et al., 2001), feature codes are assumed to be extracted from regularities in prior sensorimotor experience and can only exist by virtue of their connections to sensory codes. In the current simulation, the model contains feature codes that link to lower level sensory codes. In the same vein, feature codes link to motor codes. In our modeling we explicitly show how these associations are strengthened: through sensorimotor experience. Connections to sensory codes are grounded in regularities in sensory input; connections to motor codes are grounded in regularities in action–effects that follow motor code activation. In our model, task codes are fully generic and recruited when needed. They themselves are meaningless but only function as relay nodes when processing information from (stimulus) perception to action (effect) planning, and vice versa.

The fact that the translation from perception to action involves feature codes that are necessarily grounded in sensorimotor experience is, in HiTEC modeling, the main reason why stimulus–response congruency occurs: the model cannot perceive stimuli or plan actions without using these grounded feature codes. The feature codes used for perceiving stimuli and those used for planning actions (i.e., by anticipating and representing action–effects) are grounded in the same perceptual world (Prinz, 1992) and are therefore prone to overlap. When perception of a particular stimulus and the planning of a particular response involve the same feature code, this code overlap results in either facilitation or interference (Hommel, 2004). This is the foundation of the observed SRC effect (for a more elaborate discussion and application to a variety of SRC paradigms, see also Haazebroek et al., submitted).

By the same token, processing a task instruction is assumed to activate these feature codes grounded in sensorimotor experience. Implementing a – in principle abstract – task set automatically wires the feature codes into a stimulus–to–response processing pathway. The fact that these feature codes also represent (prior) sensorimotor experience (i.e., by virtue of their connections to sensory codes) allows the task instruction to modulate subsequent sensorimotor processing (i.e., by top–down enhancing feature codes and therefore sensory codes), even on the automatic level of SRC.

HiTEC is also compatible with the idea that concepts are flexible and context-dependent. According to the embodied cognition view, concepts are learned from recurrent sensorimotor experiences. During those experiences, the patterns of activity in sensory-motor brain areas are captured and stored in memory to form elaborated, multimodal knowledge structures, called simulators. Representation is achieved by reactivating a subset of this stored knowledge to construct a specific simulation. The exact content of a particular simulation depends on the individual’s experience with the simulated concept, as well as on situational factors such as current goals and task demands (Barsalou, 1982, 1993; van Dantzig et al., 2011). This flexibility is strongly reflected in HiTEC. For example, in the current simulation, the task instruction influenced how an ambiguous movement was encoded and represented in the model. Similarly, the context or task instruction could influence which features or feature dimensions of a stimulus are most relevant, and thereby enhance the processing of these features or dimensions (the intentional weighting principle). Indeed, several recent studies have shown that spatial congruency effects only occur when participants perform a task that emphasizes the relevant conceptual dimension of a stimulus. For example, Schubert (2005) found that spatial congruency between power and vertical position only occurred when participants made power judgments of words such as “king” or “servant,” but not when they judged the valence of these stimuli. Similarly, Zanolie and Pecher (under review) found a spatial congruency effect between number size and horizontal position when participants processed the magnitude of numbers, but not when they simply viewed the numbers or judged whether the numbers were even or uneven. Similar results were obtained by Santiago et al. (2012), who showed that conceptual congruency effects only appeared when participants attended to the relevant conceptual dimension, either through task instruction or by means of exogenous attentional cueing.

To conclude, perception, cognition, and action interact by using common representations. Many studies and theoretical accounts focus on bilateral interactions between perception–cognition, action–cognition, and perception–action. In this paper, we have shown that the interaction between perception and action is strongly influenced by cognition (i.e., task instruction). Cognition, in turn, is based on prior sensorimotor experience, and is therefore grounded in perception and action. In addition to our empirical findings on a two-dimensional Simon task we set out to provide an overarching framework that connects various findings and explains computationally *how* perception, action, and cognition interact. We hope that the combination of our empirical work and the computational model contribute to a better understanding of the complex interaction between perception, action, and cognition.

## Conflict of Interest Statement

The authors declare that the research was conducted in the absence of any commercial or financial relationships that could be construed as a potential conflict of interest.
